# *Memprot*: a program to model the detergent corona around a membrane protein based on SEC–SAXS data

**DOI:** 10.1107/S1399004714016678

**Published:** 2015-01-01

**Authors:** Javier Pérez, Alexandros Koutsioubas

**Affiliations:** a Beamline SWING, Synchrotron SOLEIL, L’Orme des Merisiers, BP 48, Saint-Aubin, 91192 Gif-sur-Yvette, France; b Jülich Centre for Neutron Science (JCNS), Forschungszentrum Jülich GmbH, Outstation at MLZ, Lichtenbergstrasse 1, 85747 Garching, Germany

**Keywords:** small-angle X-ray scattering, membrane proteins, SEC–SAXS, *Memprot*

## Abstract

The application of small-angle X-ray scattering (SAXS) to structural investigations of transmembrane proteins in detergent solution has been hampered by two main inherent hurdles. On the one hand, the formation of a detergent corona around the hydrophobic region of the protein strongly modifies the scattering curve of the protein. On the other hand, free micelles of detergent without a precisely known concentration coexist with the protein–detergent complex in solution, therefore adding an uncontrolled signal. To gain robust structural information on such systems from SAXS data, in previous work, advantage was taken of the online combination of size-exclusion chromatography (SEC) and SAXS, and the detergent corona around aquaporin-0, a membrane protein of known structure, could be modelled. A precise geometrical model of the corona, shaped as an elliptical torus, was determined. Here, in order to better understand the correlations between the corona model parameters and to discuss the uniqueness of the model, this work was revisited by analyzing systematic SAXS simulations over a wide range of parameters of the torus.

## Abstract 2

Systematic SAXS simulations have been analysed over a wide range of parameters in order to better understand the detergent corona around a membrane protein.

## 1. Introduction   

Membrane proteins are crucial for a wide range of vital functions such as transmembrane signalling, cell adhesion, molecular transport and bioenergetics. About 25% of genes encode membrane proteins (Wallin & von Heijne, 1998 ▶) and they are the target of more than 50% of modern therapeutic agents (Overington *et al.*, 2006 ▶). The difficulty in studying their structure arises in part from their tendency to aggregate when extracted from the membrane, a consequence of the hydrophobic patch on their transmembrane surface. A membrane-mimetic detergent or lipid therefore has to be included in the solutions in all steps of extraction and purification, which is often not easy to handle (le Maire *et al.*, 2006 ▶). This ­prerequisite may hinder crystallization, which is necessary for X-ray crystallographic studies, and may also complicate NMR studies owing to protein–detergent interactions (Tamm & Liang, 2006 ▶). In spite of this, the number of deposited entries in the Protein Data Bank is regularly increasing (Kozma *et al.*, 2013 ▶).

Small-angle X-ray scattering applied to biological macromolecules (BioSAXS) is a low-resolution structural technique that is particularly well adapted to monitor ternary and quaternary conformational changes of soluble proteins in their buffer solution (Koch *et al.*, 2003 ▶). Although SAXS does not provide molecular-level resolution, it is particularly accurate in distinguishing between different structural models proposed from higher resolution techniques, and has become a popular technique among protein crystallographers (Hura *et al.*, 2009 ▶). Applying SAXS to solubilized membrane proteins has long been hindered by the necessary presence of detergent molecules in solution, which leads to extra contributions to the measurable signal that are difficult to separate from the protein signal. These unwanted contributions arise from the detergent free micelles in solution and from the corona-like self-assembled detergent structure that covers the hydrophobic surface of the protein. Owing to their different chemical compositions, detergent heads and tails have quite different electron densities that are both different from the typical mean electron density of a protein molecule. The usual analysis of BioSAXS data, based on the calculation of scattering invariants or on the use of *ab initio* methods (Svergun, 1999 ▶), is not directly applicable. Even using small-angle neutron scattering (SANS), in which the contrast of different species in solution can be systematically varied by simply changing the ratio between H_2_O and D_2_O in the buffer, adequate contrast matching of the two different detergent parts can hardly be achieved (Breyton *et al.*, 2013 ▶).

In recent work, Berthaud *et al.* (2012 ▶) proposed an original strategy to obtain structural information on membrane proteins from SAXS data. Unlike previous studies on proteins with unknown structures, the goal was to properly model the detergent corona around a membrane protein of known structure. The expected consequence of this initial step is to obtain a solid structural basis for later studies of complexes between the membrane protein and other macromolecular partners or of conformational changes of the protein. In a study on the bovine eye lens major intrinsic protein aquaporin-0 (AQP0), a combination of SEC-HPLC with SAXS data collection (reviewed in Pérez & Nishino, 2012 ▶) was used to collect a scattering curve from the protein–detergent complex unbiased by the contribution of the free detergent micelles. The SAXS data were then fitted to a model of the protein–detergent complex using the atomic structure of the membrane protein and a detergent corona modelled as a coarse-grained elliptical torus with two distinct electron densities. Optimizing the fits based on this geometrical model gave parameters concerning detergent organization (the overall thickness of the detergent layer and the extent of hydrophobic/hydrophilic regions) that were in agreement with previous experimental studies on detergent micelles (Lipfert *et al.*, 2007 ▶) and with independent measurements from refractometry coupled UV absorption spectroscopy (Berthaud *et al.*, 2012 ▶). Yet, it was not stated that the actual shape of the detergent corona is indeed elliptical. In subsequent work (Koutsioubas *et al.*, 2013 ▶), we improved the interpretation of the geometrical model of the detergent corona by implementing a ‘model-free’ *ab initio* coarse-grained fitting algorithm that provided a more objective estimation of the detergent corona shape. We then showed that the corona actually tends to mimic the shape of the transmembrane contour of aquaporin-0 in a way reminiscent of the detergent structure determined by Pebay-Peyroula *et al.* (1995 ▶) in their neutron diffraction studies of OmpF porin crystals. It nevertheless appears that the elliptical shape provides the correct number of parameters that are strictly necessary to model the corona and correctly fit the experimental SAXS data. It has the double advantage of being easily tunable and providing a reasonable physically meaningful description of the actual detergent shape. We therefore consider the elliptical modelling to be an interesting basis to build upon for more complex studies where the structure of the protein of interest is only partially known.

In the present work, we revisit the geometrical approach by more thoroughly examining the correlations between all fitting parameters, and derive some rules about which strategy to adopt in further studies with different proteins. We also briefly describe the program *Memprot* that we have developed to systematize the SAXS calculations from the geometrical models and which is to be released to the community. In a subsequent development of our software, for cases in which the protein contour is less isometric than that of AQP-0, we consider developing a parameterized geometrical model of the detergent corona which adheres more closely to the actual shape of the protein. We expect the rules determined here to be sufficiently general to be applicable to these future modified coronas.

## 2. Modelling methodology   

The coarse-grained modelling based on a geometrical description of the detergent corona around a transmembrane protein has been thoroughly described in the work of Berthaud *et al.* (2012 ▶), while further insights into its validity to describe SAXS data were obtained by comparisons with *ab initio* and molecular-dynamics models (Koutsioubas *et al.*, 2013 ▶). Here, we briefly review this geometrical approach and also describe the new features that are implemented in the computer program that accompanies this paper.

**Figure 1:**
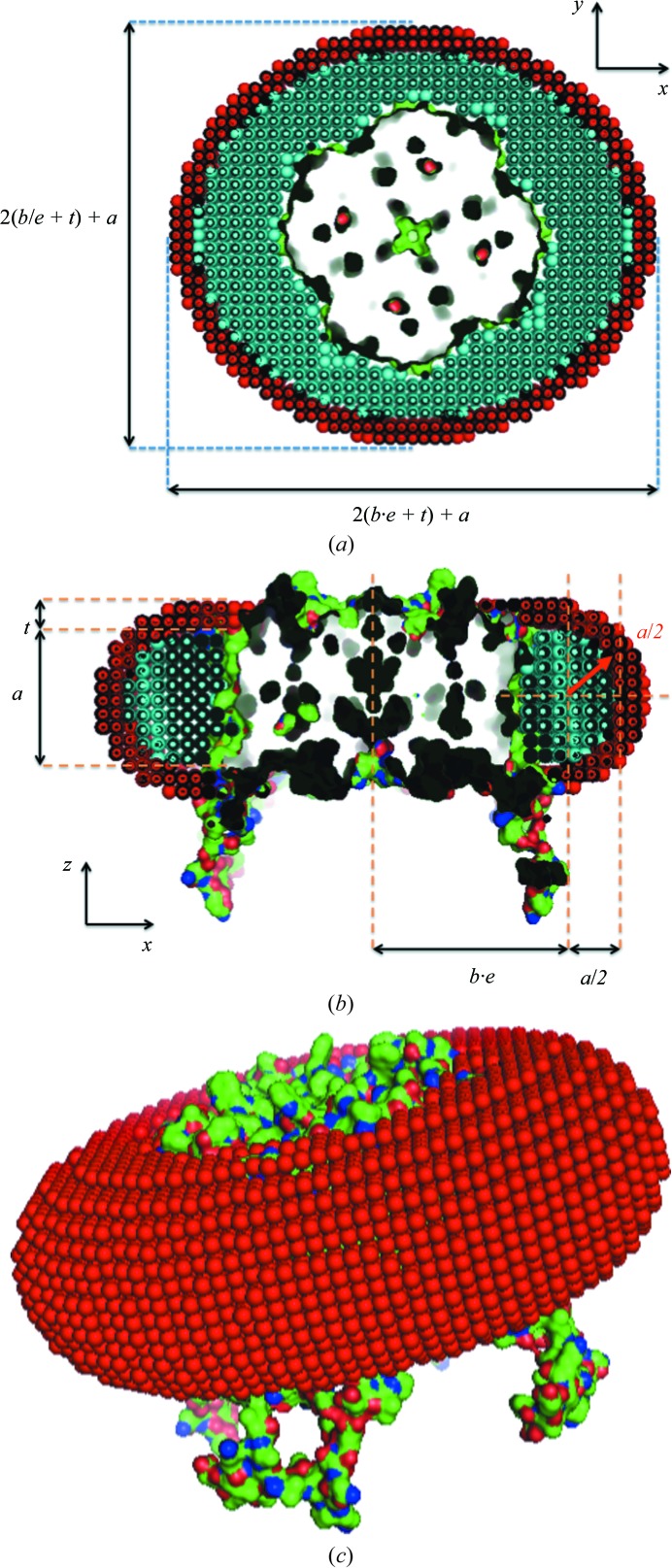
Model of the complex between the full-atom 2b6p structure and its detergent corona optimized from SEC–SAXS experimental data. (*a*) Section within the transmembrane plane. (*b*) Section perpendicular to the transmembrane plane. (*c*) Overall view.

The protein–detergent complex model is based on an all-atom representation of the open-pore version of aquaporin-0 (PDB entry 2b6p, with added residues 1 and 36, from Gonen *et al.*, 2005 ▶) and on a coarse-grained network description of the detergent corona around the hydrophobic protein surface. The different electron densities ρ of the hydrophobic and hydrophilic regions of the corona are taken into account by placing two specifically chosen types of pseudo-atoms at the nodes of two densely packed cubic networks with different network spacing. Once the models have been generated and formatted as a PDB file, the calculations of SAXS profiles are performed using the program *CRYSOL* (Svergun *et al.*, 1995 ▶). The pseudo-atoms were chosen within the *CRYSOL* look-up table, which contains for each atom its intrinsic excluded volume (*V*_vdW_) and its number of electrons (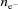). For a given electron density of the corona model, the spacing of each of the two networks of elementary cell volume (*V*_elementary cell_) are related to the previous quantities according to the relation
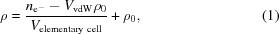
where ρ_0_ is the buffer electron density and ρ is the electron density of the specific region. Depending on the electron density of each region of the detergent corona, the pseudo-atom type was selected according to (1) so as to result in a elementary cell size of the network comparable to the excluded volume of the pseudo-atom (hydrophobic part of the corona, CH_3_ pseudo-atoms with a network parameter of about 3.1 Å; hydrophilic part of the corona, NH_3_ pseudo-atoms with a network parameter of about 2.8 Å). The hydrophobic hydrocarbon tails of the detergent that assemble around the protein surface are modelled as an elliptical hollow torus of height *a* and cross-sectional minor and major axes *b*/*e* and *b* × *e*, where *e* is the ellipticity of the torus in the *xy* plane of the membrane (Fig. 1 ▶). The torus is centred on the symmetry axis (*z*) of the protein or, in the case of lack of symmetry, around the axis that passes through the approximate centre of the transmembrane part. In turn, the hydrophilic region occupied by the detergent polar headgroups is modelled as an exterior shell of constant thickness *t* surrounding the inner hydrophobic torus.

In order to determine the set of geometric parameters that best reproduces the experimentally measured scattering *I*_exp_(*Q*), a fine search of the parameter space is performed and the model with the best agreement is selected. The agreement factor is defined as 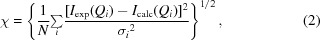
where *I*_calc_(*Q*_*i*_) and σ*_i_* are the intensity calculated with *CRYSOL* and the experimental standard deviation at *Q* = *Q*_*i*_, respectively, and *Q*_*i*_ is the momentum transfer related to the X-ray wavelength λ and to the scattering angle 2θ_*i*_ by the relation *Q*_*i*_ = 4πsinθ_*i*_/λ. During the calculation of the discrepancy between the experimental and the model scattering with *CRYSOL*, two additional parameters are left relatively free in order to obtain better fits. These are the electron-density contrast of the hydration layer around the complex and the overall excluded volume parameter α that slightly modifies the average electron densities of the protein atomic groups and of two corona regions according to the relation
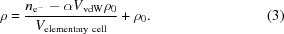
The inclusion of this extra parameter therefore changes, but only marginally, the final electron densities of the two corona regions^1^.

Compared with the previous implementation of the algorithm (Berthaud *et al.*, 2012 ▶), an additional parameter that may be scanned during the fitting procedure is the rotation ϕ of the corona axis in the *xy* plane. In cases where the protein transmembrane part is not characterized by radial symmetry (as in the case of β-barrels), variation of the in-plane rotation may help to obtain lower χ values.

## 3. Computer program   

The procedure for the fitting of SAXS data from membrane protein–detergent systems based on a geometric representation of the detergent corona has been implemented in a computer program written in Fortran 90 called *Memprot*. In order to perform a fit the user is asked for the all-atom PDB structure file of the correctly oriented protein, for the experimental curve, if possible accompanied by the error estimates, and for the range of parameter space (*a*, *b*, *t*, *e*, ϕ) that will be scanned. An initial guess about the electron density of the different parts of the corona is also needed.

**Figure 2:**
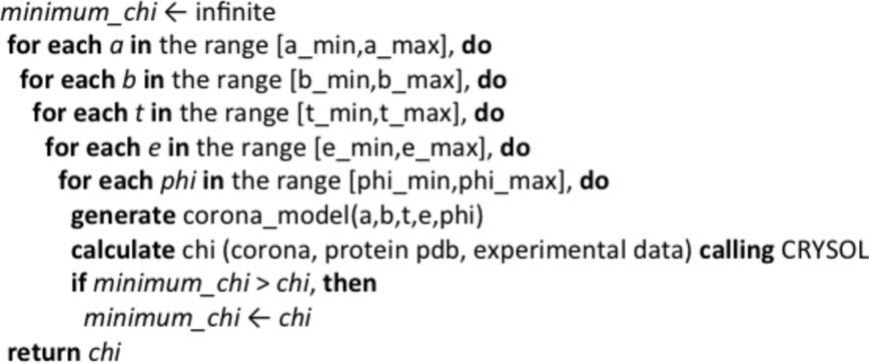
Algorithm of the *Memprot* program. The program essentially creates PDB files with the models made of the full-atom protein structure and the parameterized coarse-grained detergent corona, and *CRYSOL* is called to calculate the SAXS curves. An overall sorting on the χ value is performed to keep the best model.

The program proceeds by thoroughly scanning the parameter space (with a selected step for each parameter) in an effort to recognize the model parameters with the lowest discrepancy against the experimental data (Fig. 2 ▶). In order to ensure accuracy in the final results and also the fastest possible run time, the user is asked to limit the number of spherical harmonics *L* used by *CRYSOL* according to the relation *L* = 5 + *Q*_max_ *D*_max_/2, where *Q*_max_ is the maximum considered momentum transfer and *D*_max_ is the maximum diameter of the protein–detergent complex that may be estimated from the experimental data using *GNOM* (Svergun, 1992 ▶). The latter relation comes from a conservative estimation of the higher order Bessel function contributions as implemented in *CRYSOL* calculations.

The detergent corona models are always generated around the *z* axis and centred at *z* = 0. For this reason, a small script was developed that helps the user to align the protein along the *z* axis and also to bring the middle of the transmembrane plane to *z* = 0, resulting in a new PDB file with the protein in a suitable orientation. Furthermore, the user may also fine-tune the elevation of the protein with respect to the *xy* plane in cases in which the hydrophobic transmembrane surface is not easily identifiable.

Overall execution times depend linearly on the number of steps required for each of the parameters that are scanned. At the end of each run the user is provided with ASCII files that summarize the results and also with detailed fitting curves and PDB files for each of the trial models. The program depends on the installation of *CRYSOL*. Executables for the program are available for all major platforms (Windows, Mac and Linux).

## 4. Results and discussion   

It has been shown that the SAXS data from *n*-dodecyl-β-d-maltopyranoside (DDM)-solubilized aquaporin-0 tetramers can be fitted with great detail using the geometric elliptic toroidal model of the detergent corona (Berthaud *et al.*, 2012 ▶). The obtained fitted parameters concerning detergent organization (the overall thickness of the detergent layer and the extent of hydrophobic/hydrophilic regions) were in agreement with previous experimental studies of detergent micelles (Lipfert *et al.*, 2007 ▶) and with independent measurements from refractometry coupled UV absorption spectroscopy. The additional elaborate modelling of the data with model-free coarse-grained bead models and molecular-dynamics simulation (Koutsioubas *et al.*, 2013 ▶) elucidated the need to break the circular symmetry and the associated inclusion of ellipticity in the geometric model. Here, by performing extensive fits of the original SAXS data of the aquaporin-0–DDM system using our updated algorithm, we aim at a more comprehensive understanding of the interplay between the parameters of the model and also of the overall stability of the obtained solutions. In the following sections, we primarily focus on (i) the correlation between the geometric parameters, (ii) the effects of the corona electron densities on the best-fit parameters, (iii) the potential benefits of varying the in-plane rotation of the corona with respect to the protein and (iv) the effects of minor conformational changes of the protein structure on the final obtained models.

### 4.1. Correlation between the geometric parameters   

**Figure 3:**
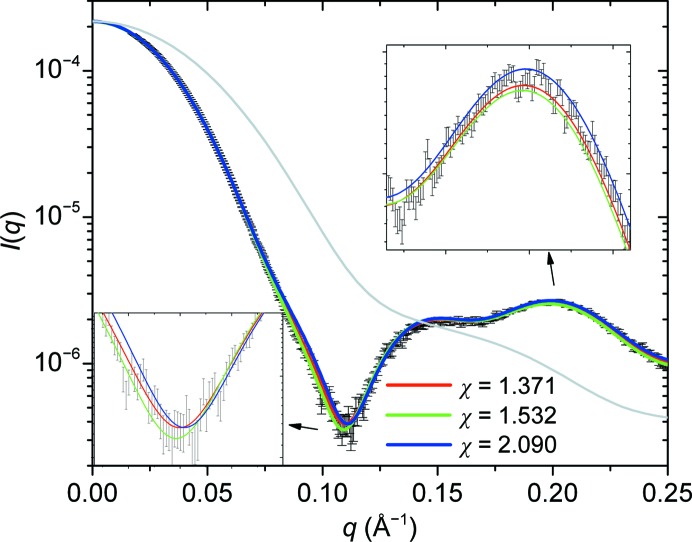
Illustration of the χ-value sensitivity. The SEC–SAXS experimental curve of AQP0 in DDM is superposed with curves calculated using three different detergent torus parameters, all three of which result in low χ values. The regions with the most pronounced discrepancies are highlighted in the two insets. It clearly appears that the slight differences between the χ values correspond to statistically meaningful differences in the curves. The curve calculated from the bare AQP-0 is shown in light grey as additional information.

**Figure 4:**
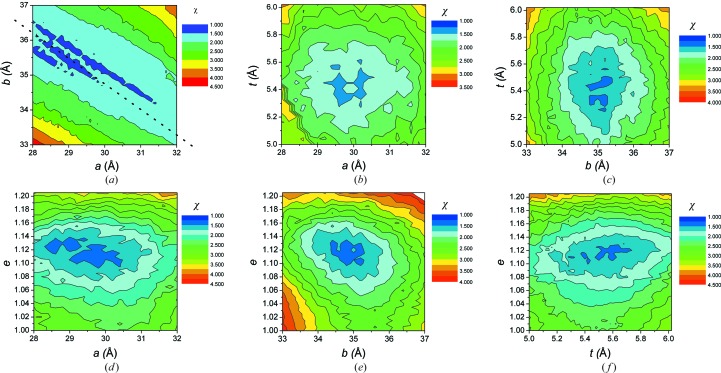
Contour plots of χ as a function of different torus parameter pairs while the remaining parameters are kept at their optimum value, as determined by Berthaud *et al.* (2012 ▶) (*a* = 30 Å, *b* = 35 Å, *t* = 5.5 Å, *e* = 1.12). See Fig. 1 ▶ for the definition of the parameters. No strong correlations between the fitting parameters appear to exist, except between the parameters *a* and *b*, which define the diameter and thickness of the corona, respectively. The dotted line in (*a*) is a guide for the eye showing the main direction of this correlation.

**Figure 5:**
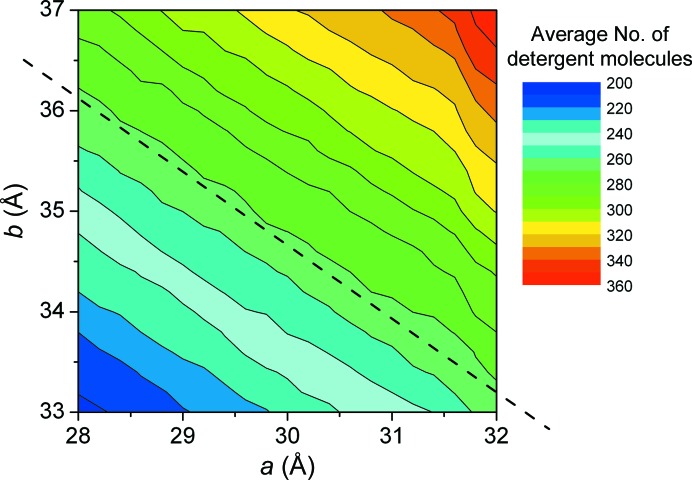
Mean number of detergent molecules as a function of the parameters *a* and *b*. The dotted line is a reproduction of the line in Fig. 4 ▶(*a*).

In total, the geometric model of the corona has four parameters that define the shape of the complex (excluding, in the present case, the in-plane rotation). This means that it is difficult in a single plot to identify the effect of the variation of each parameter on the agreement of the model with the experimental curve. For this reason, we chose to perform runs of the fitting algorithm by keeping two of the parameters close to the values where we observe the global χ minimum as determined in Berthaud *et al.* (2012 ▶) and leaving the other two parameters free. In this way, we may produce contour plots that provide insight into the dependence of the overall fit on each parameter. In Fig. 3 ▶, three curves corresponding to different models with relatively low χ values are plotted in order to visualize the impact of goodness of fit. It appears that, in our case, χ values below 1.5 lead to hardly distinguishable curves that very nicely fit the data, while a curve with a χ value of 2 more clearly departs from the experimental curve. Fig. 4 ▶ summarizes the results for each pair of parameters. For all pairs except (*a*, *b*) a well defined region of low χ values exists pinpointing a global minimum, underlying both the independence between the parameters and the uniqueness of their value. For the parameter pair (*a*, *b*), *i.e.* the lengths defining the height and the elliptical axes of the torus, respectively, extended regions of similar χ values along straight lines can be identified (see the dotted line in Fig. 4 ▶ *a*). In this small range around the optimum values, *a* and *b* are therefore correlated non-independent parameters, thus making it relatively hard to locate a global minimum of χ. The correlation between *a* and *b* appears to be given approximately by the relation *b* + *a*/2 ≅ constant. Given that the average outer radius of the hydrophobic part of the corona is close to *R* ≅ *b* + *a*/2, it might not be totally surprising that models with equal values of their radius give rise to similar values of χ. However, the parameter *a* not only influences the radius of the corona but also directly its height. We then checked how the variation of *a* and *b* influences the total volume of detergent. In Fig. 5 ▶, the number of detergent molecules directly calculated from the number of beads composing the corona is displayed for the same parameter set (*a*, *b*) as in Fig. 4 ▶(*a*). The related contour plot is shown together with the approximate iso-χ straight line found in Fig. 4 ▶(*a*). It clearly appears that this straight line is also parallel to the iso-number of detergent molecule lines or equally to regions of the *a*, *b* plane where the total complex volume is constant. In brief, the correlation between the torus diameter and the torus height observed in Fig. 4 ▶(*a*) within a narrow, although meaningful, range of values is such that the resulting number of detergent molecules is kept the same. Thus, SAXS alone appears not to provide a constraint strong enough to decorrelate these two physical entities within the abovementioned narrow range. Although not demonstrated, we anticipate that this might be generalized to any membrane-protein complex. External considerations, such as the known height of the transmembrane region of the protein or the expected length of the detergent hydrophobic tail, might then help to identify the most meaningful pair of values.

### 4.2. Effects of the electron densities of the detergent corona   

From previous experimental SAXS studies of detergent micelles in solution (Lipfert *et al.*, 2007 ▶), we have estimations of the electron densities of the hydrophilic polar and hydrophobic regions of the micelles. Assuming that the detergent organization has similar properties around the protein surface, we may expect that the same electron densities may apply. Nevertheless, it is interesting to see how small variations in the imposed electron density may affect the obtained results, also in relation to the fact that during the *CRYSOL* fitting procedure the free excluded volume parameter α also affects the final effective electron-density values.

**Figure 6:**
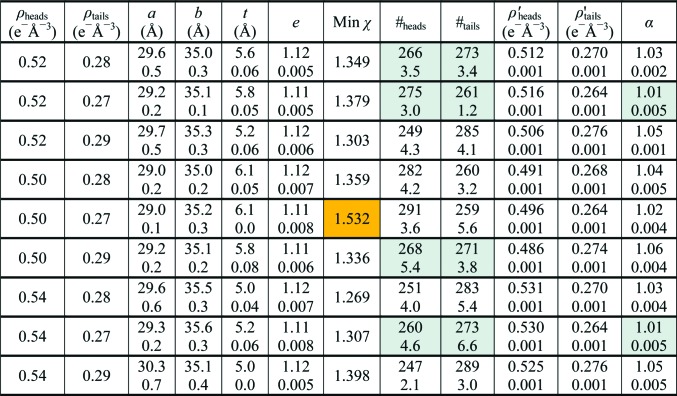
The results of multiple runs with variable given electron densities for the hydrophilic (ρ_heads_) and the hydrophobic (ρ_tails_) parts of the corona. The quantities ρ′_heads_ and ρ′_tails_ represent the final electron densities after taking into account the fitted value of the electronic contrast bias (α) by *CRYSOL*. Geometric parameters and electron densities are mean values for all models with χ within +5% difference from the global minimum, which have essentially nearly indistinguishable scattering curves. The standard deviation for each calculated quantity is indicated below the corresponding mean value. The orange shaded case indicates the worst agreement between the model and the experimental data. The grey-shaded data denote the most self-consistent results, in terms of agreement between the number of detergent molecules calculated either from the hydrophobic or the hydrophilic volumes of the model and in terms of the lowest average contrast bias.

In this respect, by executing a very fine search around the electron-density values used before, with over 2 × 10^4^ curve evaluations for each set of electron densities in the range ρ_heads_ = 0.52 ± 0.02 e^−^ Å^−3^ and ρ_heads_ = 0.28 ± 0.01 e^−^ Å^−3^, we aimed at an estimation of the stability of the obtained model parameters. The mean values of the best parameters are shown in Fig. 6 ▶, together with their standard deviation.

From this set of runs, we may evaluate the mean values and related uncertainties of the geometric parameters: *a* = 29.4 ± 0.4 Å, *b* = 35.2 ± 0.2 Å, *t* = 5.5 ± 0.4 Å and *e* = 1.115 ± 0.005. The number of detergent molecules estimated by the number of pseudo-atoms in each part of the corona is #_heads_ = 265 ± 15 and #_tails_ = 273 ± 11. As can be seen from the low standard deviations of each of these values, the optimization of the geometrical parameters appears to be quite robust and to be independent to some extent of the precise electron densities chosen to model the detergent corona. However, a closer inspection of Fig. 6 ▶ reveals that for some of the nine tested conditions (lines 1, 2, 6 and 8), despite similar χ values the agreement between the number of detergent molecules deduced from the hydrophobic volume and from the hydrophilic volume is much better than for the remaining conditions. Notably, for the cases where this agreement is good, the contrasts of the electron densities with respect to water were both decreased or increased compared with the initial values, with a positive contrast for the hydrophilic part and a negative contrast for the hydrophobic part. In contrast, for the cases showing lower agreement one of the contrasts was increased while the other was decreased. This suggests that our approach allows the determination of the ratio between the values of the electron-density contrasts of opposite sign more accurately than their actual values. We also note that the electron- density values used by Berthaud *et al.* (2012 ▶) are in the ‘good’ pool. Among this pool, only for the cases in lines 2 and 8 is the parameter α virtually equal to 1. For these two pairs of electron densities, the specific volume of the complex did not need to be artificially altered by the fitting procedure. These two cases therefore represent the most coherent models of all. It can be noted that these two cases share the same final hydrophobic electron density of 0.264 e^−^ Å^−3^.

### 4.3. In-plane rotation of the corona structure   

As already described, for aquaporin-0, which is a tetramer structure with an axis of symmetry, we do not expect the in-plane rotation to play a major role in improvement of the fitting results. However, we systematically varied ϕ in order to verify these expectations. For each in-plane rotation value we search for the parameter set that gives the lowest χ values. It appears that the fitted model parameters are almost unaffected by rotating the corona with respect to the protein, which is very probably owing to the lack of anisometry of the AQP-0 protein itself. As expected from geometrical considerations, for 0 and 90° rotation we recover essentially the same minimum χ, while for intermediate values of ϕ the fits become slightly less good, with a maximum relative increase of χ by about 10% for 60° rotation (data not shown).

### 4.4. Overall fit sensitivity to the membrane structure   

**Figure 7:**
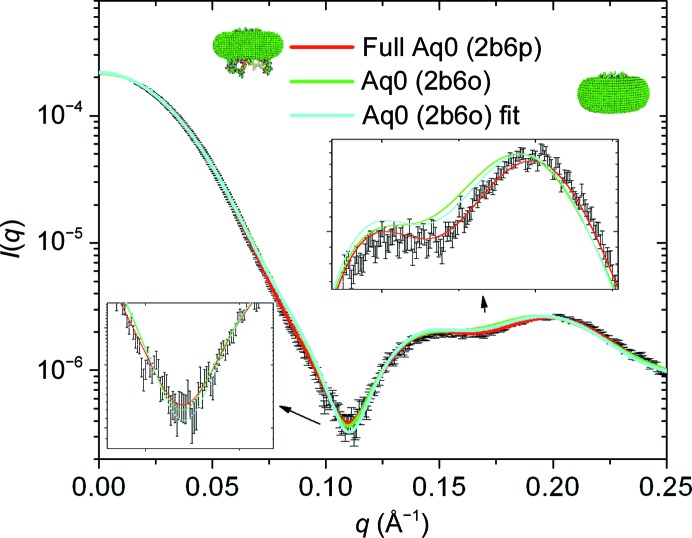
Scattering curves corresponding to corona parameters *a* = 29.6 Å, *b* = 35.4 Å, *t* = 5.6 Å, *e* = 1.12, *e*′_heads_ = 0.512 e Å^−3^, *e*′_tails_ = 0.270 e Å^−3^) for the full (2b6p) and truncated (2b6o) structures of aquaporin-0. The respective χ values are 1.31 and 3.79. The curve corresponding to an artificial optimized corona using the truncated form of aquaporin-0 is also plotted. The associated χ value is 3.47, which is still much higher than that for the complex based on the actual 2b6p structure.

As previously discussed, the methodology developed at this stage does not aim to resolve the low-resolution structure of membrane proteins of completely unknown structure. Rather, the modelling of the detergent corona may provide a route for (i) the validation of candidate structures of membrane proteins or (ii) the study of extra-membraneous conformational changes associated with the function of the protein. In this respect, it is of interest to quantify the effects of small structural modifications of the protein structure itself on the overall goodness of fit of the model. In order to do so, we chose a low-χ model of the detergent corona obtained for the full aquaporin-0 conformation (PDB entry 2b6p) and we calculated the associated scattering curve with a truncated aquaporin-0 structure with the extramembrane C-terminal domains missing (22 residues per monomer). This structure, also called the closed-pore conformation (PDB entry 2b6o, Gonen *et al.*, 2005 ▶), has the residues forming the pore slightly closer to each other than in the full aquaporin-0. From a cellular point of view, the truncated form results from the maturation process undergone by the fibrillar cells of the eye lens when they migrate from the cortex to the core of the lens (Bassnett *et al.*, 2011 ▶). As can be seen in Fig. 7 ▶, the fit of the SAXS curve calculated from the 2b6o model of aquaporin-0 associated with the corona previously optimized for the 2b6p model, with parameters *a* = 29.6 Å, *b* = 35.4 Å, *t* = 5.6 Å, *e* = 1.12, rapidly deteriorates at medium *Q* values and leads to a high value of χ. This is not surprising, as the lack of a substantial fraction of the protein structure should necessarily change the SAXS curve. More interesting is to check whether the experimental data contain enough information to prevent us from reaching a good fit with a wrong model. For this purpose, a search of the parameter space for a biased model of the corona artificially compatible with the truncated structure of aquaporin-0 was performed. The best agreement was achieved with the following parameters: *a* = 31.2 Å, *b* = 34.3 Å, *t* = 5.8 Å, *e* = 1.11. As would be expected, the values of the parameters *a* and *b* have increased to ‘compensate’ for the lack of the extracellular domains in the 2b6o model. However, the final agreement of χ ≃ 3.1 is still considerably higher than the best fit with the full structure. This means that the physical constraints imposed on the corona model appear to be strong enough to disallow the detergent–protein complex based on a wrong protein structure from fitting the data. To further check the sensitivity to discriminate between two slightly different structures, we performed the same type of calculations based on a chimera formed *in silico* from the 2b6o structure to which were added the C-terminal domains of the 2b6p structure. The resulting curve was then indistinguishable from that obtained from the full 2b6p model (not shown), which means that the structural differences in the pore region between 2b6p and 2b6o are too tiny to be discriminated.

## 5. Conclusions   

Based on previous HPLC–SAXS data for the DDM–AQP-0 complex (Berthaud *et al.*, 2012 ▶), we have attempted to quantify the degree of confidence that can be attributed to the modelling of the detergent corona around a membrane protein in solution using a parameterized geometrical description of the detergent. For this purpose, we have investigated the correlations between each of the fitting parameters. We have shown that the pool of parameters resulting in the best fit lies in a global minimum, guaranteeing the uniqueness of their values, with one single exception. The only observed correlation, between the lateral extension of the torus and its height, could be clearly linked to a constraint on the total number of detergent molecules, and a way to solve the resulting ambiguity was proposed. The validity of the electron densities that we used to model the detergent corona could be discussed after thoroughly examining the influence of their variations on the resulting models. The effect of the in-plane orientation of the detergent elliptical corona was assessed, and although not of great impact in the case of the very isotropic AQP-0, it could be of potential importance in modelling complexes of more anisometric proteins. The most striking result, showing that we were not able to fit the curve using a ‘slightly’ wrong model of the protein, was not totally expected and suggests that HPLC-coupled SAXS measurements of detergent-solubilized membrane proteins together with the presented modelling may have the ability not only to distinguish between different protein structural features and associated modifications at an intermediate resolution but also potentially to discard structural models for which no good fit can be obtained.

Until now, our approach has only been applied to AQP-0, and no conclusions of too wide a generality can be derived from a single case. It is therefore our expectation that by distributing the program used to perform the corona modelling, other such projects can be conducted and may improve our knowledge of membrane-protein structures. Having provided a protocol, we expect that other projects could follow in order to assess the quality of the conclusions.

The program *Memprot* will be distributed as an executable and will be downloadable from http://www.synchrotron-soleil.fr/Recherche/LignesLumiere/SWING.

## Footnotes

1 The excluded volume parameter α may fluctuate in the range 0.93–1.07 and the hydration layer contrast in the range 0–0.075 e Å^−3^.
